# Effect of Silicon Nitride Coating on Titanium Surface: Biocompatibility and Antibacterial Properties

**DOI:** 10.3390/ijms25179148

**Published:** 2024-08-23

**Authors:** Akina Tani, Harumitsu Tsubouchi, Lin Ma, Yurie Taniguchi, Yasuyuki Kobayashi, Mariko Nakai, Satoshi Komasa, Yoshiya Hashimoto

**Affiliations:** 1 Department of Oral Health Sciences, Faculty of Health Sciences, Osaka Dental University, 1-4-4, Makino-honmachi, Hirakata-shi 573-1121, Osaka, Japan; tani-a@cc.osaka-dent.ac.jp; 2 Department of Removable Prosthodontics and Occlusion, Osaka Dental University, 8-1, Kuzuhahanazono-cho, Hirakata-shi 573-1121, Osaka, Japan; tsubouchi-h@cc.osaka-dent.ac.jp (H.T.); ma-lin0617@outlook.com (L.M.); 3 Department of Anesthesiology, Osaka Dental University, 8-1, Kuzuhahanazono-cho, Hirakata-shi 573-1121, Osaka, Japan; taniguchi-yu@cc.osaka-dent.ac.jp; 4 Osaka Research Institute of Industrial Science and Technology, Morinomiya Center, 1-6-50, Morinomiya, Joto-ku, Osaka 536-8553, Osaka, Japan; 5 Department of Forensic Dentistry, Osaka Dental University, 8-1 Kuzuhahanazono-cho, Hirakata-shi 573-1121, Osaka, Japan; nakai-m@cc.osaka-dent.ac.jp; 6 Department of Biomaterials, Osaka Dental University, 8-1 Kuzuhahanazono-cho, Hirakata-shi 573-1121, Osaka, Japan; yoshiya@cc.osaka-dent.ac.jp

**Keywords:** implant, biocompatibility, antibacterial material, silicon nitride, titanium

## Abstract

In recent years, with the advent of a super-aged society, lifelong dental care has gained increasing emphasis, and implant therapy for patients with an edentulous jaw has become a significant option. However, for implant therapy to be suitable for elderly patients with reduced regenerative and immunological capabilities, higher osteoconductive and antimicrobial properties are required on the implant surfaces. Silicon nitride, a non-oxide ceramic known for its excellent mechanical properties and biocompatibility, has demonstrated high potential for inducing hard tissue differentiation and exhibiting antibacterial properties. In this study, silicon nitride was deposited on pure titanium metal surfaces and evaluated for its biocompatibility and antibacterial properties. The findings indicate that silicon nitride improves the hydrophilicity of the material surface, enhancing the initial adhesion of rat bone marrow cells and promoting hard tissue differentiation. Additionally, the antibacterial properties were assessed using Staphylococcus aureus, revealing that the silicon nitride-coated surfaces exhibited significant antibacterial activity. Importantly, no cytotoxicity was observed, suggesting that silicon nitride-coated titanium could serve as a novel implant material.

## 1. Introduction

Worldwide, doctors and material scientists are collaborating to accelerate and sustain the integration of dental implants into both hard and soft tissues. Additionally, they are working to expand implant coverage to improve overall implant performance [1,2,3]. The surface characteristics of implant materials significantly impact the rate and degree of osseointegration. Surface-modifying compounds have been shown to enhance osteogenic differentiation, cell adhesion, and growth significantly [4]. These compounds can maintain the secondary stability of the bone and promote implant integration [5,6,7]. The quick and direct integration of bone into an implant, known as osseointegration, plays a significant role in the functional loading of implants. Various methods and techniques have been developed to enhance implant stability and osseointegration [8,9]. For instance, the chemical and mechanical abrasion of implant surfaces has been shown to significantly improve bone bonding compared to untreated surfaces [10,11,12]. These treatments are believed to promote the adsorption of fibronectin or other proteins onto the implant surface, which stimulates osteoblasts to form focal adhesions through an integrin-mediated process [13,14,15].

Titanium, a biocompatible material widely used in implants, naturally forms a thin oxide coating on its surface that is highly resistant to corrosion. Numerous surface modification methods for titanium have been explored, focusing on the surface characteristics of biomaterials to improve the osseointegration of dental implants [16,17,18,19].

Despite advancements in implant therapy, peri-implantitis remains a growing concern in dental practice. Peri-implantitis, an inflammatory lesion, is caused by bacteria from periodontal disease infecting the area around the implant [20,21,22,23,24]. Similar to chronic periodontitis, chronic infections are characterized by peri-implant bone loss, peri-implant mucosal edema, and redness [25,26,27,28]. The implant body affected by peri-implantitis often harbors bacteria commonly found in chronic periodontitis. The development of a biofilm on the implant surface is influenced by oral bacteria present during implantation, allowing bacteria to colonize the newly implanted body through biofilm formation in the subgingival plaque and periodontal pockets of the remaining teeth [29,30]. Therefore, current implant material surface research is focused on the requirements of significant hard tissue differentiation ability and antimicrobial qualities [31,32].

Silicon nitride ceramics have been applied as in vivo implants due to their high heat resistance, corrosion resistance, mechanical properties, and fracture toughness [33,34,35]. Silicon nitride has been reported to exhibit osteoconductivity in vivo and is expected to be applied as a dental implant material [36,37]. Recently, silicon nitride has demonstrated antibacterial properties, with studies by Gorth et al. indicating that silicon nitride promotes osteogenesis and inhibits bacterial adhesion around the implant [38].

However, the mechanism by which silicon nitride surfaces influence bone formation and bacterial adhesion inhibition is still unclear, and it will take time before silicon nitride alone can be widely used as an implant material. Consequently, we explored the possibility of creating an implant material with high osteogenic and antibacterial properties by depositing silicon nitride on titanium surfaces. Implant treatments with silicon nitride deposition have potential clinical applications, including immediate treatment prior to implant placement and the treatment of peri-implantitis.

## 2. Results

### 2.1. Surface Characterization

**Figure 1:**
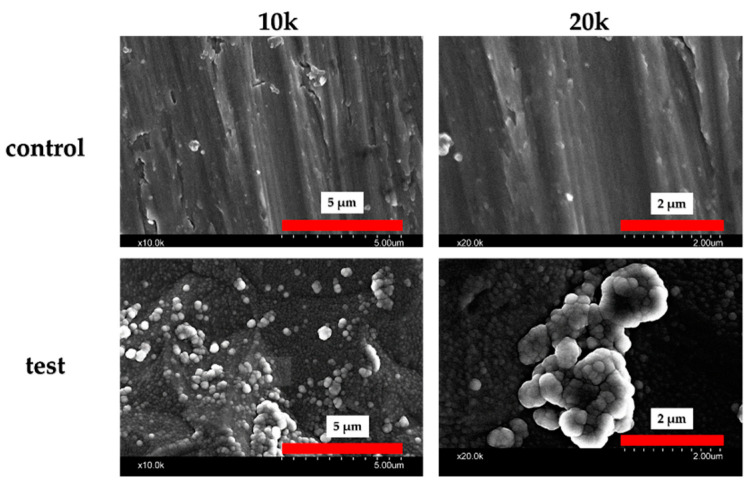
SEM analysis of the control and test groups. Silicon nitride crystal deposition was observed on the surface of the material in the experimental group.

**Figure 2:**
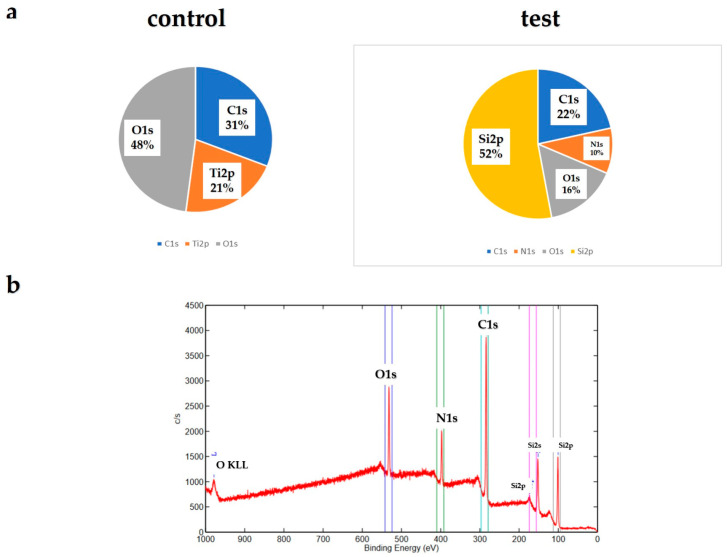
XPS analysis of the control and test groups (**a**). The XPS spectra of a wide scan of the test group (**b**). The presence of Si and N, the main elements of silicon nitride, was observed on the material surfaces of the experimental group, while Ti was not detected.

**Figure 3:**
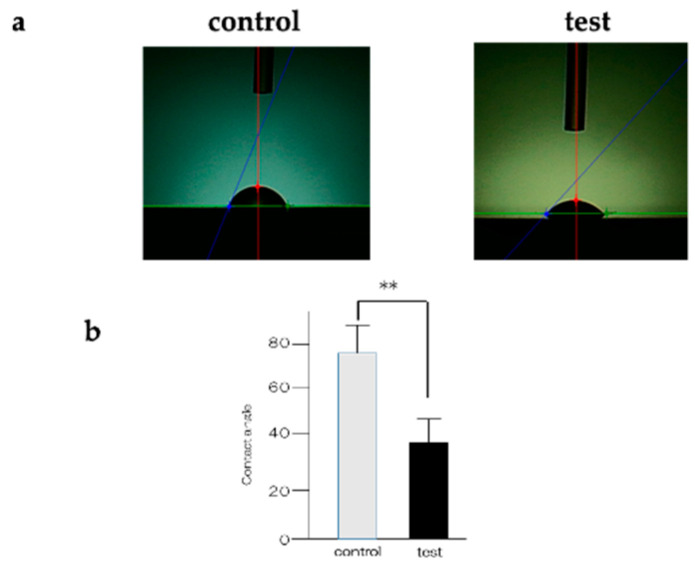
Contact angle analysis of the control and test groups (**a**). A decrease in contact angle was observed in the experimental group. The contact angle of the test group was lower than that of the control group (*n* = 4, ** *p* < 0.01) (**b**).

An assessment of the titanium samples is shown in Figure 1. The material in the experimental group formed crystalline layers, which were verified by Scanning Electron Microscope (SEM) studies. Numerous spherical crystalline accumulations were observed at high magnification on the surfaces of the materials in the experimental group. Elemental analysis using X-ray photoelectron spectroscopy (XPS) (Figure 2) detected silicon, nitrogen, oxygen, and carbon molecules on the surface of the materials in the experimental group. The presence of these elements indicates that silicon nitride was deposited on their surfaces. Contact angle evaluation using distilled water showed a decrease in the contact angle in the experimental group (Figure 3).

### 2.2. An Evaluation of Protein Adsorption on the Test and Control Titanium Surfaces

**Figure 4:**
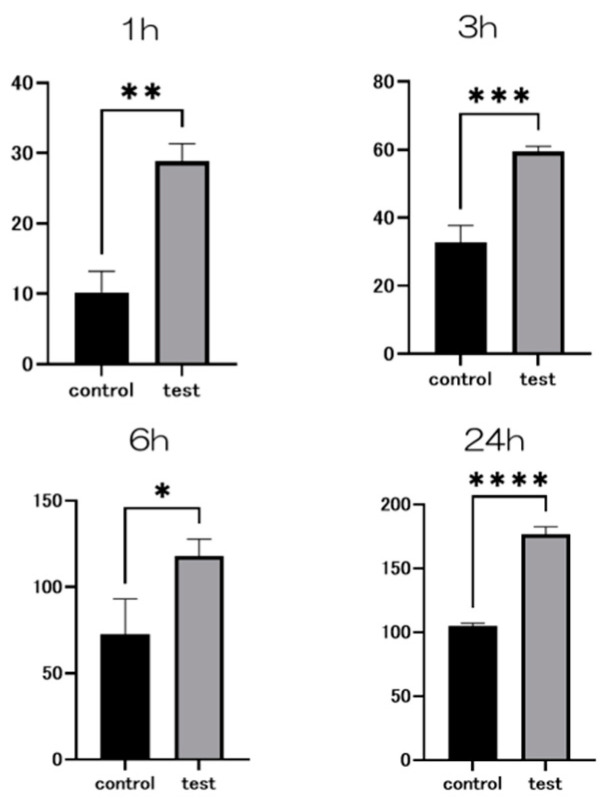
The results of the BSA adhesion examination showing significantly higher adsorption in the test group compared to the control group (*n* = 4, * *p* < 0.1, ** *p* < 0.01, *** *p* < 0.001, **** *p* < 0.0001).

The adhesion examination findings for each of the test and control groups are presented. The test group exhibited significantly higher bovine serum albumin (BSA) adsorption compared to the control group (Figure 4).

### 2.3. Effects of SiN-Coated Titanium Surface on Cell Adhesion and Morphology in Rat Bone Marrow Cells (RBMCs)

**Figure 5:**
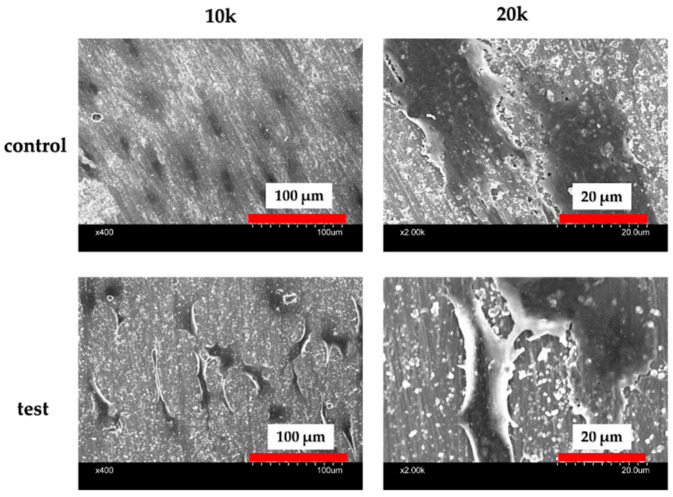
Fluorescence microscope observations of RBMCs’ morphology on the titanium surface after 6 h of culture. RBMCs’ adhesion to the surface of the materials was confirmed for both groups.

**Figure 6:**
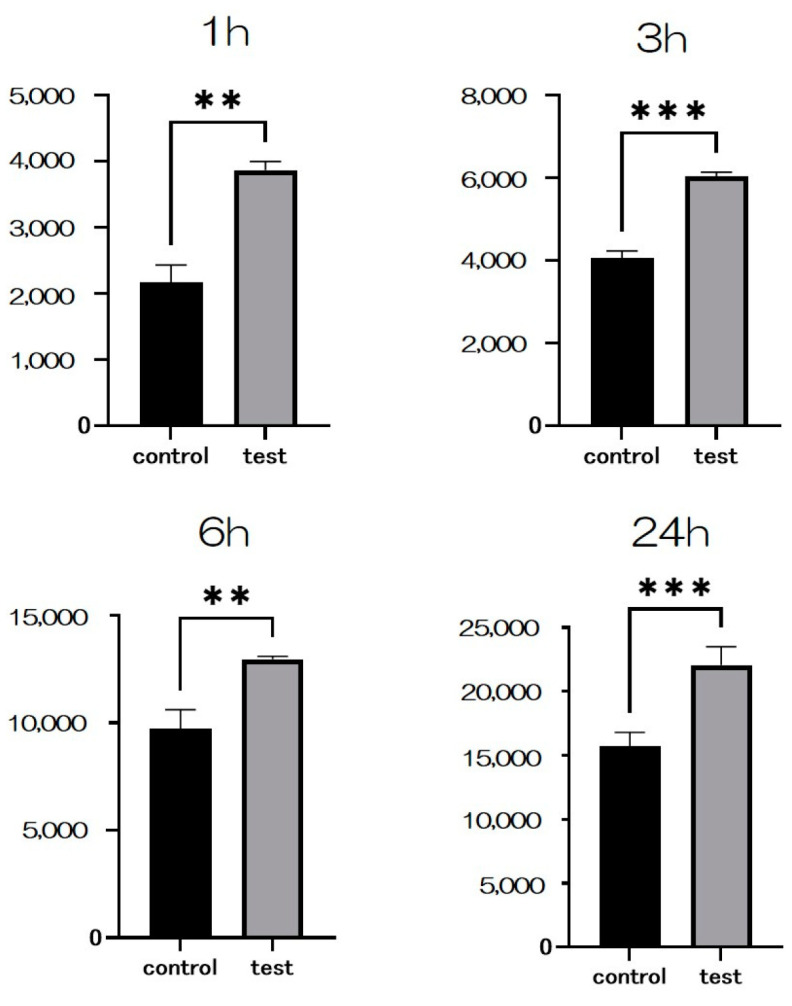
After Si_3_N_4_ coating on the material surface, the number of adhered RBMCs was statistically significantly higher than that in the control group (*n* = 4, ** *p* < 0.01, *** *p* < 0.001).

An SEM was employed to observe the morphology of rat bone marrow cells (RBMCs) on the titanium surface after 6 h of culture. It was confirmed that the RBMCs adhered to the surface of both groups. Following silicon nitride (SiN) coating on the material surface, the number of adhered RBMCs was significantly higher compared to the control group (Figure 5). Throughout all measurement time points, the test group exhibited notably greater RBMC adhesion numbers than the control group (Figure 6).

### 2.4. SiN Coating Method Induced Bone Differentiation on Titanium Surface In Vitro

**Figure 7:**
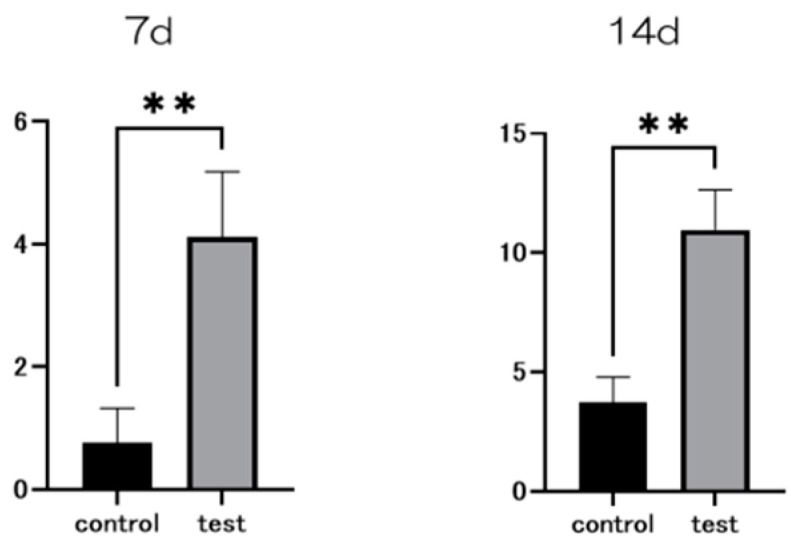
Alkaline phosphatase (ALP) expression in bone marrow cells at days 7 and 14 after the start of the culture was significantly higher on the material surface of the test group compared to the control group (*n* = 4, ** *p* < 0.01).

**Figure 8:**
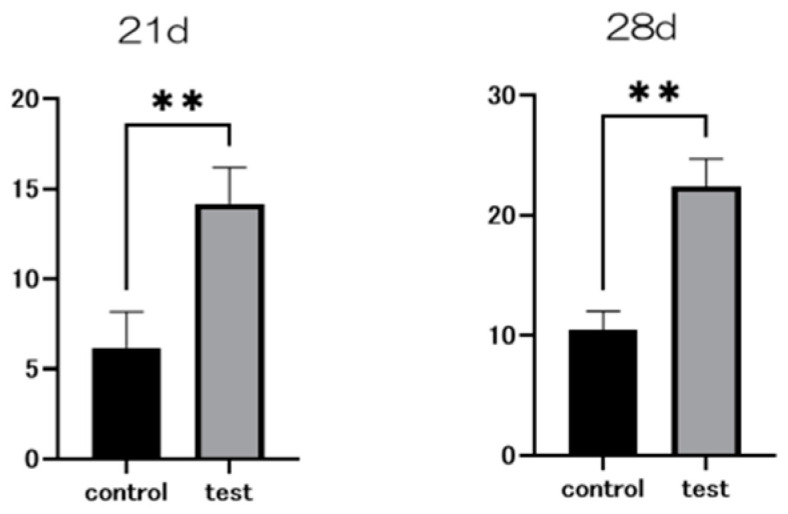
Calcium deposition in bone marrow cells at days 21 and 28 after the start of the culture was significantly higher on the material surface of the test group compared to the control group (*n* = 4, ** *p* < 0.01).

**Figure 9:**
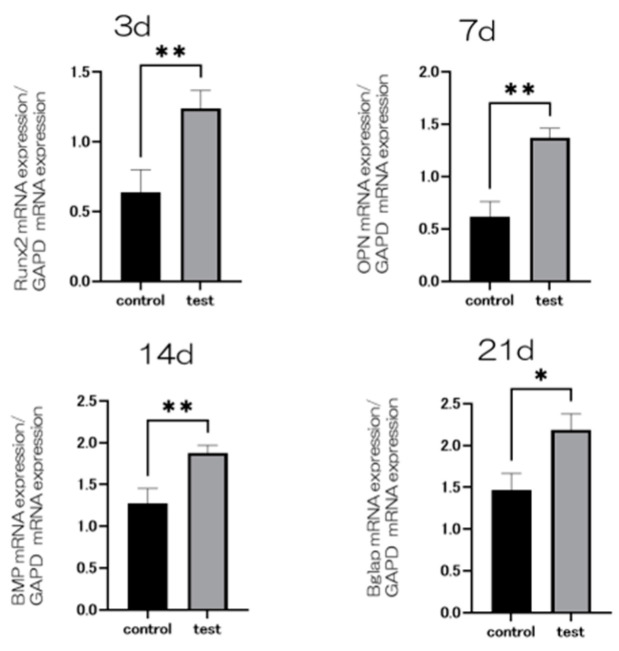
Gene expression related to the induction of hard tissue differentiation was analyzed on the material surface of samples from the test and control groups. The assay was performed at specific measurement times for each gene. Significantly higher gene expression was observed on the material surface of the test group compared to the control group at all measurement time points (*n* = 4, * *p* < 0.1, ** *p* < 0.01).

At seven and fourteen days after the start of the culture, the material surface of the test group showed significantly higher levels of alkaline phosphatase (ALP) expression compared to the control group (Figure 7). Similarly, at 21 and 28 days post-culture, the test group’s material surface exhibited significantly greater calcium deposition than that of the control group. Gene expression related to the induction of hard tissue differentiation was evaluated on the material surfaces of both test and control groups (Figure 8). The assay was conducted at specific time points unique to each gene. Notably, gene expression on the material surface of the test group was consistently higher than that of the control group (Figure 9).

### 2.5. Antibacterial Activity

**Figure 10:**
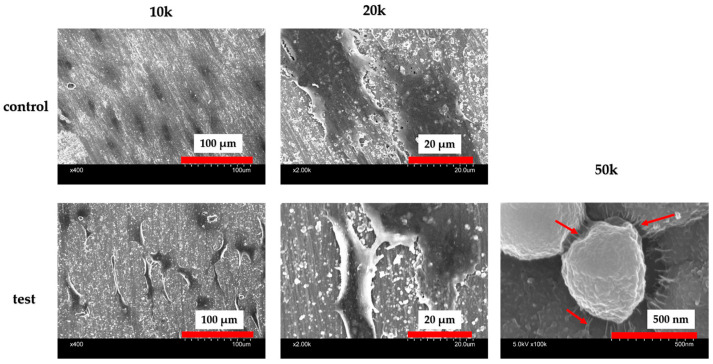
SEM images of the material surfaces of the test and control groups seeded with Staphylococcus aureus are depicted. It is evident that there was minimal bacterial adhesion to the surface of the experimental group. Furthermore, at high magnification, bacteria were observed to be attached to the silicon nitride crystals via pseudopodia (red arrow).

**Figure 11:**
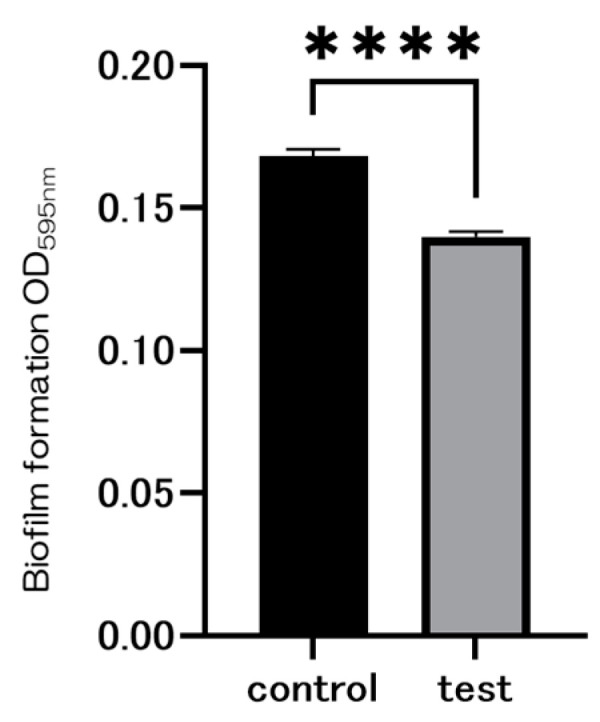
The results of the bacterial adhesion test using Staphylococcus aureus biofilms are presented. Significantly lower values were observed in the test group compared to the control group (*n* = 4, **** *p* < 0.0001).

The figure presents SEM observations of bacterial adherence on the material surfaces. The control group exhibited a significant amount of bacterial adhesion, whereas the experimental group showed minimal adhesion (Figure 10). Figure 11 compares the biofilm quantities formed, demonstrating a substantial decrease with the SiN coating.

### 2.6. Cytotoxicity Test

**Figure 12:**
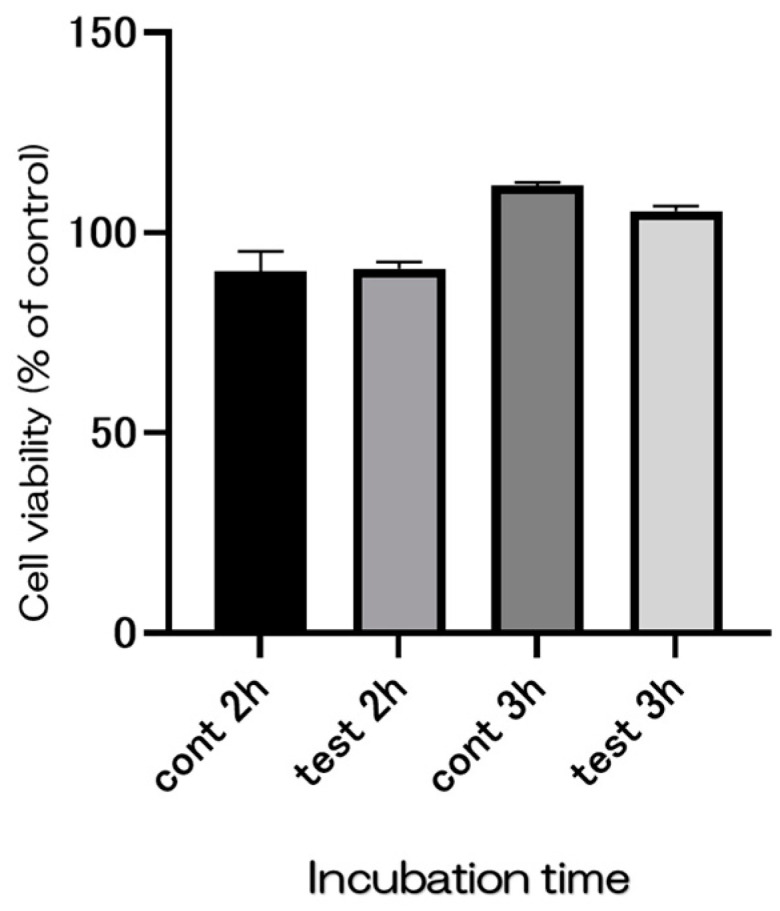
The evaluation of cytotoxicity using V79 cells. The material surface did not affect cell growth in both the test and control groups.

Figure 12 illustrates the results of the cytotoxicity tests conducted on the test samples using absorbance measurements. V79 cells were cultured to evaluate the impact of the test sample’s extract, obtained using a growth medium, on cell viability. There was no significant difference in viability between SiN-coated titanium and the control group.

## 3. Discussion

The objective of this study was to investigate the effects of silicon nitride deposition on pure titanium metal surfaces on tissue response surrounding implant placement at the in vitro level, focusing on the induction of hard tissue differentiation and antibacterial properties. SEM and XPS analyses confirmed silicon nitride deposition on the titanium surface using the plasma chemical vapor deposition (PCVD) method. In vitro evaluation with rat bone marrow cells demonstrated improved initial cell adhesion and an enhanced ability to induce hard tissue differentiation on the silicon nitride-deposited titanium surface. Additionally, antibacterial activity assessed with Staphylococcus aureus showed reduced bacterial adhesion on the silicon nitride-deposited titanium surface. These results highlight the material’s potential as a novel implant material with strong capabilities in inducing hard tissue differentiation and possessing antimicrobial properties.

Silicon nitride, deposited in this study, is an advanced material widely utilized across various industries due to its exceptional properties. Composed of silicon and nitrogen, silicon nitride ceramics offer a unique combination of strength, hardness, and thermal stability [34,35,36,37]. In the medical field, these ceramics are employed in orthopedic implants such as joint replacements and dental implants, owing to their biocompatibility, low friction, and high wear resistance, making them suitable for long-term implantation in the human body. The ability of silicon nitride to promote bone growth and osseointegration further enhances its utility in orthopedics. Various methods are available for the deposition of silicon nitride on titanium surfaces. In consultation with Tohokaken, we used the PVCD method, which can deposit highly reactive and dense silicon nitride. SEM analysis revealed silicon nitride crystals on the surface of the materials in the experimental group, indicating increased surface roughness, while a decreased contact angle was also observed. According to the paper reported by Ryan et al., it is clear that the contact angle decreases with an increase in the nitrogen layer, and in this experiment, it is also clear that the contact angle decreases with the deposition of silicon nitride [39]. These improvements in surface characteristics likely contributed to the enhanced adhesion of bone marrow cells and proteins involved in inducing hard tissue differentiation. Moreover, the presence of nitrogen observed in XPS analysis has been linked to the promotion of hard tissue differentiation in previous studies. Overall, silicon nitride deposition on titanium surfaces shows promising performance characteristics required for dental implant materials [40].

Upon implantation, protein coatings covered the surfaces of all implant materials, regulating the reaction and behavioral cascade of bone marrow cells [41]. The most prevalent plasma protein, albumin, exhibited the highest absorption rate in the argon plasma-treated group in this experiment, surpassing the control group. Additionally, plasma treatment enhanced albumin absorption compared to the untreated control group. Furthermore, the Si_3_N_4_ coating on titanium surfaces improved cell adhesion, including osteoblasts and fibroblasts. Thus, changes in surface energy may encourage tissue growth by enhancing the adsorption of specific proteins compared to materials with microscale features.

RBMCs on titanium metal coated with Si_3_N_4_ exhibited increased ALP activity, calcium deposition, and expression of bone formation-related genes compared to RBMCs on the surface of the untreated control group. ALP activity indicates early-stage bone differentiation, bone production, and osteoblast activity [42]. The expression levels of osteoblast-specific markers were significantly different on the surface of titanium implants covered with Si_3_N_4_ compared to the untreated titanium surfaces. Furthermore, the Si_3_N_4_-coated surfaces upregulated Bglap, Runx2, BMP-2, and ALP in RBMCs, supporting stromal cell differentiation into osteoblasts while preserving their vitality.

Although numerous implant materials show potential for enhancing hard tissue differentiation, the prevalence of peri-implant inflammatory conditions and their associated treatment challenges remain significant concerns [24,25,26,27,28,29]. Antimicrobial dental implants offer distinct advantages over traditional materials, potentially mitigating the financial and health-related burdens of peri-implant disease. Previous research has explored incorporating chemical antibacterial agents or silver nanoparticles into titanium surfaces, yet their long-term viability remains uncertain. Nevertheless, the matter at hand pertains to their long-term sustainability. Although more implant materials may be able to better induce hard tissue differentiation, the high incidence of peri-implant inflammatory illness and the challenges associated with its treatment are concerning. Antimicrobial dental implants have several advantages over traditional materials and can help reduce the financial and health-related costs associated with peri-implant disease. Previous investigations have demonstrated the integration of chemical antibacterial agents or silver nanoparticles into pure titanium metal. However, the issue pertains to long-term sustainability. Consistent with the experimental results, the hydrophilicity of the material surface was assumed to be responsible for these results. In the SEM images, the SI_3_N_4_ crystals and Staphylococcus aureus were observed as if they were linked. Considering that bacterial growth was not observed in the experimental group, it is possible that silicon nitride acted directly on the bacteria, which requires further investigation. Some studies suggest that silicon nitride promotes the production of peroxynitrite in bacterial cells and is associated with the ability to lyse bacteria, consistent with the results observed in the present SEM images. Silicon nitride has been suggested that it has a strong ability to absorb proteins. Because silicon nitride is amorphous in nature, it is thermodynamically unstable in oxidative or humid environments, degrading bacteria. Therefore, the results of this paper utilizing silicon nitride deposited on oxidized titanium may be guided by the following reasons. Moreover, this study found no evidence of cytotoxicity when silicon nitride was applied to titanium surfaces, suggesting potential benefits for implant survival rates and patient outcomes, thereby improving overall implant prognosis and patient quality of life.

## 4. Materials and Methods

### 4.1. Sample Preparation

Grade 2 titanium disks (15 mm in diameter and 1 mm thick; Daido Steel, Osaka, Japan) were prepared initially. These disks were then polished sequentially using SiC abrasive sheets with grit sizes of 800, 1000, and 1500. In the experimental group, silicon nitride thin films were deposited using the plasma chemical vapor deposition process at a low substrate temperature (<60 °C). Gas irradiation was performed with the cooperation of Tohokaken Co., Ltd. (Tohokaken Co., Ltd., Saitama, Japan). The deposited silicon nitride film is 200 nm thick.

### 4.2. Surface Characterization

An SEM (S-4000; Shimadzu, Kyoto, Japan) was utilized to qualitatively assess the surface topography of the samples. X-ray photoelectron spectroscopy (XPS) analysis (Kratos Analytical Axis Ultra DLD electron spectrometer; Kratos Instruments, Manchester, UK) was conducted to determine the composition of the coatings using a monochromatic Al Kα X-ray source. Prior to analysis, each sample underwent argon ion etching for two minutes at an evaporation rate of five nanometers per minute to remove surface impurities. Elemental analysis was performed using Multipak software (Multipakv9.6.1; ULVAC-PHI, Kanagawa, Japan), applying the Shirley background and relative sensitivity coefficients provided by the instrument manufacturer. The contact angle measurements of the titanium surface were performed using a video contact angle measurement system (SImage Entry 6; Excimer Inc., Kanagawa, Japan).

### 4.3. Protein Adsorption

The model protein used was bovine serum albumin. Each specimen received 300 microliters of a protein solution (1 mg/mL protein in saline) pipetted onto it. Non-adherent proteins were extracted and combined with bicinchoninic acid (fraction V, Pierce Biotechnology, Rockford, IL, USA) at 37 °C for 1 h after incubation periods of 1, 3, 6, and 24 h. The amount of extracted albumin and the total amount of inoculated albumin were measured using a microplate reader set at 562 nm. The percentage of albumin adsorbed onto the specimens relative to the total amount was used to calculate the albumin adsorption rate.

### 4.4. Cell Culture

Rat bone marrow cells (RBMCs) were harvested from the femurs of eight-week-old Sprague-Dawley rats (SHIMIZU Laboratory Supplies Co., Kyoto, Japan). The extraction protocols followed were consistent with our previous research. Animal experiments were conducted with approval from the Medical Ethics Committee at Osaka Dental University, Japan, in accordance with ethical guidelines outlined in the National Animal Care Guidelines (approval no. 23–11001). After extraction, the RBMCs were seeded into 24-well tissue culture plates (BD Biosciences, Franklin Lakes, NJ, USA) containing titanium disks from each of the three groups at a density of 4 × 10^4^ cells per well.

### 4.5. Cell Adhesion and Morphology

Two groups’ titanium surfaces were seeded with RBMCs at an initial density of 4 × 10^4^ cells/cm^2^, and the cells were allowed to adhere to the surface for 1, 3, 6, and 24 h. The quantity of RBMC adhesion was assessed using the CellTiter-Blue^®^ Reagent (50 μL diluted in 250 μL PBS), following the manufacturer’s guidelines.

After 6 h of culture, the cells were stained and examined according to the protocol outlined in our previous publication. For SEM analysis of extracellular morphology and pseudopodia, the cell samples were processed as follows: After 24 h of incubation, the culture media were removed from the 24-well plates. The cells underwent three washes with PBS and were fixed in 1 mL of 4% PFA solution for two hours at 4 °C.

Following removal of the PFA solution, the cells were washed three times with PBS and dehydrated using a graded series of ethanol solutions (80%, 90%, 80%, 60%, and anhydrous) for 10 min each. Subsequently, they were immersed in 3-methylbutyl acetate for 30 min (S-4800; Hitachi, Ibaraki, Japan), dried in a critical-point drier (HCP-1; Hitachi), and coated with Os using an ion-sputtering machine (HPC-20; Vacuum Device, Shinkuu device, Ibaraki, Japan) for SEM examination.

### 4.6. Real-Time Reverse Transcription PCR, Alkaline Phosphatase Activity, DNA Content, and Mineralization Determination

A real-time TaqMan RT-PCR assay (Life Technologies, Carlsbad, CA, USA) was employed to quantify the expression of genes associated with osteogenesis. Total RNA was extracted using the RNeasy Mini Kit (Qiagen, Venlo, The Netherlands), and the PrimeScript RT Reagent Kit (TaKaRa Bio, Shiga, Japan) was used to reverse transcribe each 10 μL aliquot of RNA into cDNA. The mRNA expression levels of osteopontin (OPN) were assessed on day 21, bone morphogenetic protein (BMP) on day 14, and runt-related transcription factor (Runx2) on day 3, serving as markers for osteogenesis.

After 7 and 14 days of incubation, the samples were rinsed with PBS, and adherent cells were lysed in 300 μL of 0.2% Triton X-100 to measure ALP activity. The alkaline phosphatase luminometric enzyme-linked immunosorbent assay (ELISA) kit (Sigma-Aldrich, St. Louis, MO, USA) was used according to the manufacturer’s instructions to quantify ALP activity. DNA content was determined using the PicoGreen dsDNA analysis kit (Invitrogen/Life Technologies, Carlsbad, CA, USA), and the amount of DNA in each cell lysate was used to normalize ALP levels.

Calcium accumulation in the extracellular matrix was evaluated after 21 and 28 days of incubation by dissolving samples in 10% formic acid. The calcium concentration was quantified using the Calcium E-test Kit (Wako Pure Chemical Industries Ltd., Osaka, Japan) following the manufacturer’s instructions.

### 4.7. Antibacterial Activity

Tissue culture medium (TSA) and trypticase soy broth (TSB) were utilized to cultivate Staphylococcus aureus (ATCC 12600; American Type Culture Collection, Manassas, VA, USA). A single colony was selected and incubated overnight at 37 °C in 10 mL TSB. The bacterial suspension was adjusted to a concentration of approximately 1 × 10^9^ CFU/mL by diluting it in fresh TSB. To assess biofilm formation and adherence, S. aureus seed cultures were prepared and diluted to optical densities of 0.1 and 1.0, then seeded onto discs and incubated for 6–24 h at 37 °C. An SEM was used to observe bacterial adherence on different material surfaces. Subsequently, the discs were washed with PBS, stained with 2 mL of 0.05% *w*/*v* crystal violet dye for 20 min at room temperature, rinsed three times with PBS to remove excess dye, and transferred to a new 12-well plate. They were then destained for 20 min at room temperature with rotary shaking in 1 mL of 95% ethanol to quantify biofilm formation. After destaining, 100 μL of ethanol was added to each well, and the absorbance at 595 nm was measured using a SpectraMax M5 96-well microplate reader (Molecular Devices Ltd., Sunnyvale, CA, USA).

### 4.8. Cytotoxicity Test

The cytotoxicity test was conducted using the MTT method, which employs colorimetry to assess cell viability. V79 Chinese hamster cells from the JCRB cell bank were used for this assay. High-glucose DMEM supplemented with L-glutamine, phenol red, sodium pyruvate, and 10% fetal bovine serum (Bovogen Biologicals Pty Ltd., Keilor East, Australia) was obtained from FUJIFILM Wako Pure Chemical Corp., Osaka, Japan. For the test and control samples, sterile glass vials were used, each containing 1 mL of culture medium. These vials were then incubated at 37 °C for 24 h to prepare the test solutions.

### 4.9. Statistical Analyses

All findings were computed using GraphPad Prism 8.0 software (GraphPad Prism, San Diego, CA, USA) and were presented as means with standard deviations. The Student’s *t*-test was used to examine the data and make group comparisons. At *p* < 0.05, the differences were deemed statistically significant.

## 5. Conclusions

The findings of this study clearly demonstrate that the deposition of silicon nitride on the surface of pure titanium metal significantly enhances the initial adhesion of rat bone marrow cells and promotes the induction of hard tissue differentiation. Moreover, it was also found that the deposition of silicon nitride on the surface of pure titanium metal contributes to the improvement in the initial adhesion of rat bone marrow cells and the induction of hard tissue differentiation.

## Data Availability

Data are contained within the article.
